# Phenolic composition of ten plants species used as ethnoveterinary medicines in Omusati and Kunene regions of Namibia

**DOI:** 10.1038/s41598-022-25948-y

**Published:** 2022-12-09

**Authors:** N. Eiki, T. G. Manyelo, Z. M. Hassan, S. L. Lebelo, N. A. Sebola, B. Sakong, M. Mabelebele

**Affiliations:** 1 Department of Agriculture and Animal Health, College of Agriculture and Environmental Sciences, University of South Africa, Florida, 1710 South Africa; 2 Department of Life and Consumer Sciences, College of Agriculture and Environmental Sciences, University of South Africa, Florida, 1710 South Africa

**Keywords:** Plant sciences, Medical research

## Abstract

The therapeutic benefits of phenolic compounds found in plants are well known. The purpose of this study was to determine the phenolic content of ten plant species used as ethnoveterinary treatments in Namibia’s Omusati and Kunene regions. The plants of concern were *Aloe esculenta*, *Fockea angustifolia, Boscia albitrunca, Combretum imberbe, Acacia nilotica, Colophospermum mopane, Acacia erioloba, Ziziphus mucronata, Ximenia americana*, and *Salvadora persica*. An LC–MS approach was used to identify the compounds. To analyse high-resolution UPLC-UV/MS, a Waters Acquity ultra-performance liquid chromatograph (UPLC) with a photodiode array detector was connected to a Waters Synapt G2 quadrupole time-of-flight mass spectrometer (MS). The current study identified a total of 29 phenolic compounds. Flavonoids (epicatechin, (-)-Epigallocatechin, and rutin,) were the most abundant, followed by 2R, 3S-Piscidic acid. Methylisocitric acid was found in all species investigated, with the highest quantities in *A. esculenta* and *X. americana* leaf extracts. There were differences in composition and quantity of phenolic compounds in aerial and ground sections between species. The overall findings of the present study would act as a standard for subsequent investigations into the pharmacological potentials of plants species utilized as ethnoveterinary remedies. Priority should be given to isolating, purifying, and defining the active compounds responsible for these plants' activity.

## Introduction

In both industrialized and developing nations worldwide, plants are currently used as traditional veterinary treatments^1,2^. Because they are so crucial to conventional veterinary treatment, they are therefore promising candidates for drug development^3^. Secondary metabolites such as phenolic compounds, with bioactive qualities are produced by plants^4^. They are commonly found in fruits, vegetables, herbs, roots, leaves, and seeds, and they play important roles in structure, innate defence, reproduction, and sensorial properties (colour, bitterness, taste, and flavour)^5^. Phenolic compounds are released in response to UV radiation, pathogen and parasite infection, and exposure to extreme temperatures^6^.

Pentose phosphate, shikimate, and phenylpropanoid pathways produce phenolic chemicals, which are secondary metabolites^7^. The name "phenol" refers to a chemical structure that contains a phenyl ring with one or more hydroxyl substituents, and it is the most prevalent secondary metabolite in plants^8^. Phenolic substances are made up of more than one aromatic ring and one or more hydroxyl functional groups^9^. Phenolic compounds are a diverse category of molecules that have a variety of chemical configurations and are classified as monomeric, dimeric, or polymeric phenolics^10^.

The huge numbers of natural compounds, usually referred to as specialized (or secondary) metabolites, that are produced by plants are a useful resource for contemporary pharmacy^7^. Thus, the study of bioactive compounds, extracts, and new ingredients from natural sources is currently receiving a lot of attention^11^. To date, epidemiological evidence suggests that phenolic compounds have important roles, such as suppressing infections, and that they may help to reduce the occurrence of animal diseases^12^. Phenolic compounds are also thought to play an important role in the treatment of veterinary diseases as natural antioxidants, antimicrobials, and antiparasitic agents^13^. The goal of this study was to identify phenolic compounds in ten plant species claimed to be effective in treating livestock ailments like mastitis, skin infections, coughs and diarrhoea by ethnoveterinary practitioners in Namibia's Omusati and Kunene areas. These plants were *Aloe esculenta* Leach (Aloaceae); *Fockea angustifolia* K. Schum (Apocynaceae) *Boscia albitrunca* Burch. (Capparaceae); *Combretum imberbe* Wawra (Combretaceae); *Acacia nilotica* (L.) Wild*, Colophospermum mopane* (J.Kirk ex Benth.)*,* and *Acacia erioloba* E.Mey (Fabaceae); *Ziziphus mucronata* Willd (Rhamnaceae)*, Ximenia americana* L. (Olacaceae), and *Salvadora persica* L. (Salvadoraceae).

## Methods

### Ethical approval

The study was reviewed and approved by Research and Ethical Review Committee of the University of South Africa (UNISA) (approval number 2020/CAES_HREC/025). The department of forestry in Windhoek, Namibia, also gave permission for the collection of plant specimens.

### Study area

The research was conducted in Namibia's Omusati and Kunene administrative regions, which are two of the country's 13 administrative regions. Omusati and Kunene regions have a subtropical climate with hot summers and moderate winters^14^. The average annual temperature is 23–34 °C, with annual rainfall ranging from 480 to 600 mm^15^. Omusati is 796 km away from Windhoek, and Kunene is 480 km. Omusati lies at 18.4070° S and 14.8455° E, and Kunene is at 19° 24′ 31.0680″ S and 13° 54′ 51.8400″ E^16^.

### Collection and processing of plant materials

All the extracts used in this study were gathered in Namibia from plants in their natural environments. *Aloe esculenta* (leaves), *Salvadora persica* (stem bark), *Fockea angustifolia* (roots), *Boscia albitrunca* (leaf and tuber), *Combretum imberbe* (root and leaf), *Acacia nilotica* (gum and branch), *Colophospermum mopane* (leaf and bark), *Ziziphus mucronata* (leaf and root), and *Ximenia americana* (leaf) were among the plant parts harvested. These plant parts were harvested during the dry season, between August and November 2020. Mr Teodor Kaambu from the Directorate of Forestry in Windhoek in cooperation with the Namibian Botanical Research Institute (NBRI) identified the plant species employed in the current study. In taxonomic identification, the "Namibian Plants Red Data Book"^17^ was used. The Namibia Botanical Research Institute (NBRI) and international regulations prohibiting the collection of whole plants for identification purposes were followed in the collection of these plant parts. ''No complete plants were used or obtained as specimens for the current investigation. The collection of plant parts was done in accordance with Namibian laws, which are outlined in the instructions for collecting plant specimens from the Namibia Botanical Research Institute (NBRI), as well as with international laws that forbid the gathering of complete plants for identification. To avoid the transfer of pests and diseases from one location to another, as required by both Namibian and international legislation governing the collection and/or movement of plant specimens, collected plant parts were inspected for symptoms of diseases and pests''. The plant parts were air-dried for at least three weeks at room temperature before being pounded into a fine powder. The specimens were subsequently taken to Stellenbosch University in South Africa for phenolic compound analysis.

### Phenolic compound analysis

To analyse high-resolution UPLC-UV/MS, a Waters Acquity ultra-performance liquid chromatograph (UPLC) with a photodiode array (PDA) detector was connected to a Waters Synapt G2 quadrupole time-of-flight (QTOF) mass spectrometer (MS) as described by Hassan et al.^18^. Negative electrospray ionization was employed with a cone voltage of 15 V, desolvation temperature of 275 °C, desolvation gas at 650 L/h, and the remainder of the MS settings optimized for optimal resolution and sensitivity^19^.

Data was collected by scanning in resolution mode and MSE mode from 150 to 1500 m/z^20^. Two channels of MS data were recorded in MSE mode, one with a low collision energy (4 V) and the other with a collision energy ramp (20–60 V) to obtain fragmentation data. For accurate mass determination, leucine enkaphalin was utilized as the lock mass (reference mass), and the device was calibrated with sodium formate^20^. A Waters HSS T3, 2.1 100 mm, 1.7 m column was used to separate the samples as described by New et al.^21^. The mobile phase comprised 0.1% formic acid (solvent A) and acetonitrile containing 0.1% formic acid as solvent B, with a total injection volume of 2 L.

The gradient began with 100% solvent A for 1 min and then shifted to 28% B in a linear fashion over 22 min. It then moved to 40% B for 50 s, then 1.5 min at 100% B, followed by 4 min of re-equilibration to initial conditions. The column temperature was kept at 55 °C and the flow rate was 0.3 mL/min. Compounds were measured according to a calibration curve created by infusing a variety of catechin standards ranging from 0.5 to 100 mg/L catechin^22^. The chemicals used were formic acid (Merck, SA), acetonitrile 200 and methanol 215 (Romil), Catechin (Sigma Aldrich), Millipore water purification.

### Data analysis

Data was analysed using Principal component analysis (PCA) analysis was conducted using PAST version 4.02, a software for scientific data analysis with functions for data manipulation, plotting, univariate, and multivariate statistics analysis.

## Results

The study revealed the presence of 29 phenolic compounds in ten plants studied, with significant differences between species (Tables 1, 2 and 3). In *A. nilotica* branch extract for example, the primary compound was 2R,3S-Piscidic acid, while in *Z. mucronata* leaf extract, the main compound was 3-Methyl. Furthermore, corilagin and pedunculagin were only identified in *C. imberbe* leaves and root extracts, respectively. Except for *A. esculenta*, epicatechin was found in all plant species. Methylisocitric acid was identified in all species studied, except for *F. angustifolia* (root) and *A. nilotica* (gum) extracts. Dihydrox was identified only in *B. albitrunca* (leaf), *C. imberbe* (root), and *Z. mucronata* (root) extracts. Only *C. imberbe* (leaf), *X. American* (leaf), and *Z. mucronata* (root) extracts contained theogallin. Peduncu, on the other hand, was only identified in extracts from two species: *C. mopane* (bark) and *C. imberbe* (leaf and root).

**Table 1:** Medicinal properties of the plant species investigated.

Family and scientific name	Local name	Voucher number	Part used in EVM	Veterinary uses suggested	Phenolic compounds detected
Aloaceae *Aloe esculenta*	Endombo (Osh) Otjindombo (Otj) Koregu (Kkg)	KHAW2020NE	Leaf	Skin infections and coughs	Methyliso, L-Tryptophan
Apocynaceae *Fockea angustifolia*	Enongo (Osh)	KUNW2020NE	Root	Anthrax	Gallic acid, Methyliso, alpha-D-Rhap-(1- > 3)-alpha-D-Rhap, 3-Methyl (3-Methylglutaconic acid), gentesic, Panto, 1-O-vanill, L-Tryptophan,
Capparaceae *Boscia albitrunca*	Omukuzi (Osh) Omungwidi (Otj) !Hunis (Kkg)	OMUW2020NE	Tuber	Helminths	dihydrox, Gallic acid, Methyliso, alpha-D-Rhap-(1- > 3)-alpha-D-Rhap, Tachioside, 3-Methyl, gentesic, Piscidic, Panto, 6''-O-p-Co, Stizolobat, (-)-Epigallo (-)-Epigallocatechin, 1-O-vanill, Caffeic acid, L-Tryptophan, Aralidioside
			Leaf	Lung and liver infections	Gallic acid, Methyliso, alpha-D-Rhap-(1- > 3)-alpha-D-Rhap, 4,6carboxy (4,6-O-[(1R)-1-carboxyethylidene]-alpha-D-galactose), Tachioside, gentesic, Piscidic, Panto, 6''-O-p-Co, (-)-Epigallo (-)-Epigallocatechin, 1-O-vanill, Caffeic acid, L-Tryptophan, Aralidioside
Combretaceae *Combretum imberbe*	Omukuku (Osh) Omumborombonga (Otj) ǃHāb (Kkg)	OMUW2020NE	Root	Eye infection and diarrhoea	beta-Glu, dihydrox, Gallic acid, Methyliso, alpha-D-Rhap-(1- > 3)-alpha-D-Rhap, Punicalin, Tachioside, Pedunculagin, gentesic, Piscidic, Panto, 6''-O-p-Co, Peduncu, Stizolobat, (-)-Epigallo (-)-Epigallocatechin, 1-O-vanill, Caffeic acid, L-Tryptophan
			Leaf		beta-Glu, Gallic acid, Methyliso, alpha-D-Rhap-(1- > 3)-alpha-D-Rhap, Theogallin, Punicalin, Tachioside, Theogallin, Pedunculagin, Corilagin, 4-hydroxy, 3-Methyl (3-Methylglutaconic acid), gentesic, Piscidic, Panto, Peduncu, Stizolobat, (-)-Epigallo (-)-Epigallocatechin, 1-O-vanill, Caffeic acid, L-Tryptophan
Fabaceae *Acacia erioloba*	Omuthiya (Osh) Omumbonde (Otj) ǁGanab (Kkg)	KUNW2020NE	Bark	Wound healing	Gallic acid, Methyliso, alpha-D-Rhap-(1- > 3)-alpha-D-Rhap, 4,6carboxy (4,6-O-[(1R)-1-carboxyethylidene]-alpha-D-galactose), 4-Fumaryl Fumaryl (4-Fumarylacetoacetic acid), 3-Methyl (3-Methylglutaconic acid), gentesic, Panto, 6''-O-p-Co, Stizolobat, (-)-Epigallo, Isorhamn, 1-O-vanill, L-Tryptophan, Aralidioside
Fabaceae *Acacia nilotica*	Omutjuula (Osh) Olufu (Otj)	OMUW2020NE	Gum	Eye inflammation	Tachioside, 3-Methyl, Piscidic,
			Branch	Retained placenta	Gallic acid, Methyliso, alpha-D-Rhap-(1- > 3)-alpha-D-Rhap, Tachioside, 3-Methy (3 Methylglutaconic acid) l, Piscidic, (-)-Epigallo (-)-Epigallocatechin, 1-O-vanill
Fabaceae *Colophospermum mopane*	Omusati (Osh) Omuṱati (Otj) ǁGâis (Kkg)	OMUW2020NE	Leaf	Diarrhoea	beta-Glu, Gallic acid, Methyliso, 4,6carboxy, Tachioside, 4-Fumaryl, 3-Methyl, gentesic, Piscidic, Panto, 6''-O-p-Co, (-)-Epigallo (-)-Epigallocatechin, 1-O-vanill, Caffeic acid, L-Tryptophan, Aralidioside
			Bark		beta-Glu, Gallic acid, Methyliso, 4,6carboxy, Tachioside, 4-Fumaryl Fumaryl (4-Fumarylacetoacetic acid), 4-hydroxy, 3-Methyl, gentesic, Piscidic, Panto, Peduncu, (-)-Epigallo, 1-O-vanill, Caffeic acid, L-Tryptophan, Aralidioside
Olacaceae *Ximenia americana*	Omupeke (Osh) Omuninga (Otj) Blousuurpruim (Afr)	OMUW2020NE	Leaf	Eye infection and wound healing	beta-Glu, dihydrox, Gallic acid, Methyliso, alpha-D-Rhap-(1- > 3)-alpha-D-Rhap, Theogallin, 4,6carboxy, Tachioside, Theogallin, 4-Fumaryl Fumaryl (4-Fumarylacetoacetic acid), 4-hydroxy, 3-Methyl (3 Methylglutaconic acid), gentesic, Piscidic, Panto, (-)-Epigallo, 1-O-vanill, Menisdau, Hibiscitrin, L-Tryptophan, Aralidioside
Rhamnaceae *Ziziphus mucronate*	Omusheshete (Osh) Omukaru (Otj) #Aros (Kkg)	OMUW2020NE	Leaf	Diarrhoea Mastitis	beta-Glu, Gallic acid, Methyliso, alpha-D-Rhap-(1- > 3)-alpha-D-Rhap, Tachioside, 4-hydroxy, 3-Methyl (3 Methylglutaconic acid), gentesic, Piscidic, Panto, 6''-O-p-Co, (-)-Epigallo, Isorhamn, 1-O-vanill, Caffeic acid, L-Tryptophan
			Root		dihydrox, Gallic acid, Methyliso, alpha-D-Rhap-(1- > 3)-alpha-D-Rhap, Theogallin, 4,6carboxy, Tachioside, 4-Fumaryl Fumaryl (4-Fumarylacetoacetic acid), 4-hydroxy, 3-Methyl, gentesic, Piscidic, Panto, 6''-O-p-Co, Isorhamn, 1-O-vanill, Caffeic acid, L-Tryptophan, Aralidioside
Salvadoraceae *Salvadora persica*	Omunkavu (Osh) Omungambu (Otj) Khoris (Kkg)	KUNW2020NE	Bark	Skin infection	Gallic acid, Methyliso, alpha-D-Rhap-(1- > 3)-alpha-D-Rhap, 3-Methyl (3 Methylglutaconic acid), gentesic, Panto, 1-O-vanill, L-Tryptophan

**Table 2:** Phenolic compounds detected in the examined species.

Phenolic compounds	Plant species and parts screened														
	*A* *esculenta* (leaves)	*A. nilotica* (gums)	*A. nilotica* (branch)	*B. albitrunca* (tuber)	*B. albitrunca* (leaves)	*C. mopane* (leaves)	*C. mopane* (bark)	*C. imberbe* (root)	*C. imberbe* (leaves)	*A. erioloba* (bark)	*F. angustifolia* (roots)	*X. americana* (leaves)	*Z. mucronata* (roots)	*Z. mucronata* (leaves)	*S. persica* (bark)
Beta-glu	-	-	-	-	-	3	1	4	38	-	-	164	-	1	-
Dihydroxy	-	-	-	30	-	-	-	4	-	-	-	-	5	-	-
Gallic acid	-	-	15	4	10	1	1	64	71	4	-	225	9	3	1
Methyliso	63	-	3	5	3	1	2	1	2	2	-	26	1	4	1
alpha-D-Rhap-(1- > 3)-alpha-D-Rhap	-	-	1	4	2	-	-	533	6	6	-	4	265	8	1
Theogallin	-	-	-	-	-	-	-	-	1	-	-	489	1	-	-
4,6carboxy	-	-	-	-	1	108	30	-	-	1	-	1	1	-	-
Punicalin	-	-	-	-	-	-	-	7	166	-	-	-	-	-	-
Tachioside	-	62	6	4	11	3	15	59	54	-	4	12	8	3	-
Theogallin	-	-	-	-	-	-	-	-	1	-	-	745	-	-	-
Pedunculagin	-	-	-	-	-	-	-	43	120	-	-	-	-	-	-
Corilagin	-	-	-	-	-	-	-	-	87	-	-	-	-	-	-
4-Fumaryl	-	-	-	-	-	352	67	-	-	5	-	1	4	-	-
4-hydroxy	-	-	-	-	-	-	1	-	1	-	-	2	108	426	-
3-Methyl	-	1	1	2	-	1	1	-	1	2	1	3	315	1185	1
Gentesic	-	-	-	1	21	110	111	1	33	34	-	39	5	22	6
Piscidic	-	11	2903	23	2	2	2	7	4	-	-	1	2	19	-
Panto	-	-	-	6	18	1	1	12	93	7	2	47	5	3	8
6''-O-p-Co	-	-	-	1	1	1	-	148	-	10	-	-	23	1	-
Peduncu	-	-	-	-	-	-	1	3	378	-	-	-	-	-	-
Stizolobat	-	-	-	2	-	-	-	2	2	346	1	-	-	2	-
(-)-Epigallo		-	22	36	25	1	1	604	2	35	-	3	265	420	-
Isorhamn	-	-	-	-	-	-	-	-	-	247	-	-	-	-	-
1-O-vanill	-	-	113	93	228	20	29	4	1	90	12	4	15	22	1
Menisdau	-	-	-	-	-	-	-	-	-	-	-	483	-	-	-
Caffeic acid	-	-	-	6	110	119	8	1	94	-	-	-	2	2	-
Hibiscitrin	-	-	-	-	-	-	-	-	-	-	-	377	-	-	-
L-Tryptophan	1	-	68	8	29	101	68	11	136	63	-	28	24	62	46
Aralidioside	-	-	-	2	21	49	296	-	-	30	-	1	11	-	-
Total (29)															

(-) = Absence.

Beta-Glu; dihydrox; 6- {[2-(3,4-dihydroxyphenyl, Methyliso; Methylisocitric acid, Rhap-(1- > 3); alpha-D-Rhap-(1- > 3)-alpha-D-Rhap, 4,6-O-[(1R)-1-carboxy; 4,6-O-[(1R)-1-carboxyethylidene]-alpha-D-galactose. 4-Fumaryl; 4-Fumarylacetoacetic acid, 4-hydroxy; 4-hydroxy-2-oxoheptanedioate, 3-Methyl; 3-Methylglutaconic acid, Piscidic; (2R,3S)-Piscidic acid, Panto; Pantothenic acid, 6''-O-p-Co; 6''-O-p-Coumaroyltrifolin, Peduncu; Pedunculagin, (-)-Epigallo; (-)-Epigallocatechin, Isorhamn; Isorhamnetin 3-(6''-p-coumarylglucoside), 1-O-vanill; 1-O-vanilloyl-beta-D-glucose, Menisdau; Menisdaurin, Glucosyring; Glucosyringic acid; Syringin 4-O-beta-glucoside.

**Table 3:** Compounds detected in ten plant species used as ethnoveterinary medicines in Omusati and Kunene regions of Namibia.

Peak	Compound	[M-H] elemental composition	[M-H] formula	Retention time (min)
1	Beta-glu	C_18_H_32_O_16_	331,067	5.49
2	Dihydroxy	C_3_H_6_O_3_	609,127	5.65
3	Gallic acid	C_7_H_6_O_5_	169,014	5,69
4	Methyliso	C_4_H_5_NOS	205,034	5.84
5	alpha-D-Rhap-(1- > 3)-alpha-D-Rhap	C_18_H_32_O_13_	309,118	6.00
6	Theogallin	C_14_H_16_O_10_	343,064	6,53
7	4,6 carboxy	C_9_H_5_F_2_NO_2_	249,062	6.66
8	Punicalin	C_34_H_22_O_22_	781,053	6.767
9	Tachioside	C_13_H_18_O_8_	301,094	6.90
10	Theogallin	C_14_H_16_O_10_	343,064	7.10
11	Pedunculagin	C_34_H_24_O_22_	783,062	7.13
12	Corilagin	C_27_H_24_O_18_	633,069	7.35
13	4-Fumaryl	C_8_H_8_O_6_	199,025	7.59
14	4-hydroxy	C_6_H_12_O_2_	187,024	7.70
15	3-Methyl	C_5_H_10_O	143,034	7.73
16	Gentesic	C_7_H_6_O_4_	315,071	7.84
17	Piscidic	C_11_H_12_O_7_	255,051	8.01
18	Panto	C_16_H_14_F_2_N_3_NaO_4_S	218,103	8.02
19	6''-O-p-Co	C_30_H_26_O_13_	593,132	8.06
20	Peduncu	C_18_H_16_O_7_	783,070	8.12
21	Stizolobat	C_9_H_9_NO_6_	226,035	8.17
22	(-)-Epigallo	C_22_H_18_O_11_	305,065	8.26
23	Isorhamn	C_31_H_28_O_14_	623,140	8.33
24	1-O-vanill	C_8_H_8_O_3_	329,085	8.49
25	Menisdau	C_14_H_19_NO_7_	312,106	8.77
26	Caffeic acid	C_9_H_8_O_4_	355,066	9.11
27	Hibiscitrin	C_8_H_10_O_7_	495,073	9.24
28	L-Tryptophan	C_11_H_12_N_2_O_2_	203,082	9.34
29	Aralidioside	C_18_H_24_O_13_	447,114	9.49

The content of phenolic compounds in aerial and ground sections differed substantially. The leaf extract of *Z. mucronata*, for example, contained beta-Glu compound, whereas the root did not Theogallin, 4,6carboxy (4,6-O-[(1R)-1-carboxyethylidene]-alpha-D-galactose), 4-Fumaryl (4-Fumarylacetoacetic acid), and Aralidioside were found in the root extract of the same plant, but not in the leaf extract. Similarly, stizolobat and 3-Methyl (3-Methylglutaconic acid) were exclusively found in the tuber extract of *B. albitrunca*, but not in its leaf extract. Each phenolic compound's content in aerial and ground sections of the same species varies significantly. The quantity of (-)-epigallo ((-)-Epigallocatechin) and 3-Methyl (3-Methylglutaconic acid) in *Z. mucronata* leaf extract were higher than those in its root extract. The leaf extract of *C. mopane* had more 4,6carboxy (4,6-O-[(1R)-1-carboxyethylidene]-alpha-D-galactose) and 4-Fumaryl (4-Fumarylacetoacetic acid) than the bark extract. Tachioside concentrations in the same species' leaf extracts, on the other hand, were lower than those in the bark extracts.

**Figure 1:**
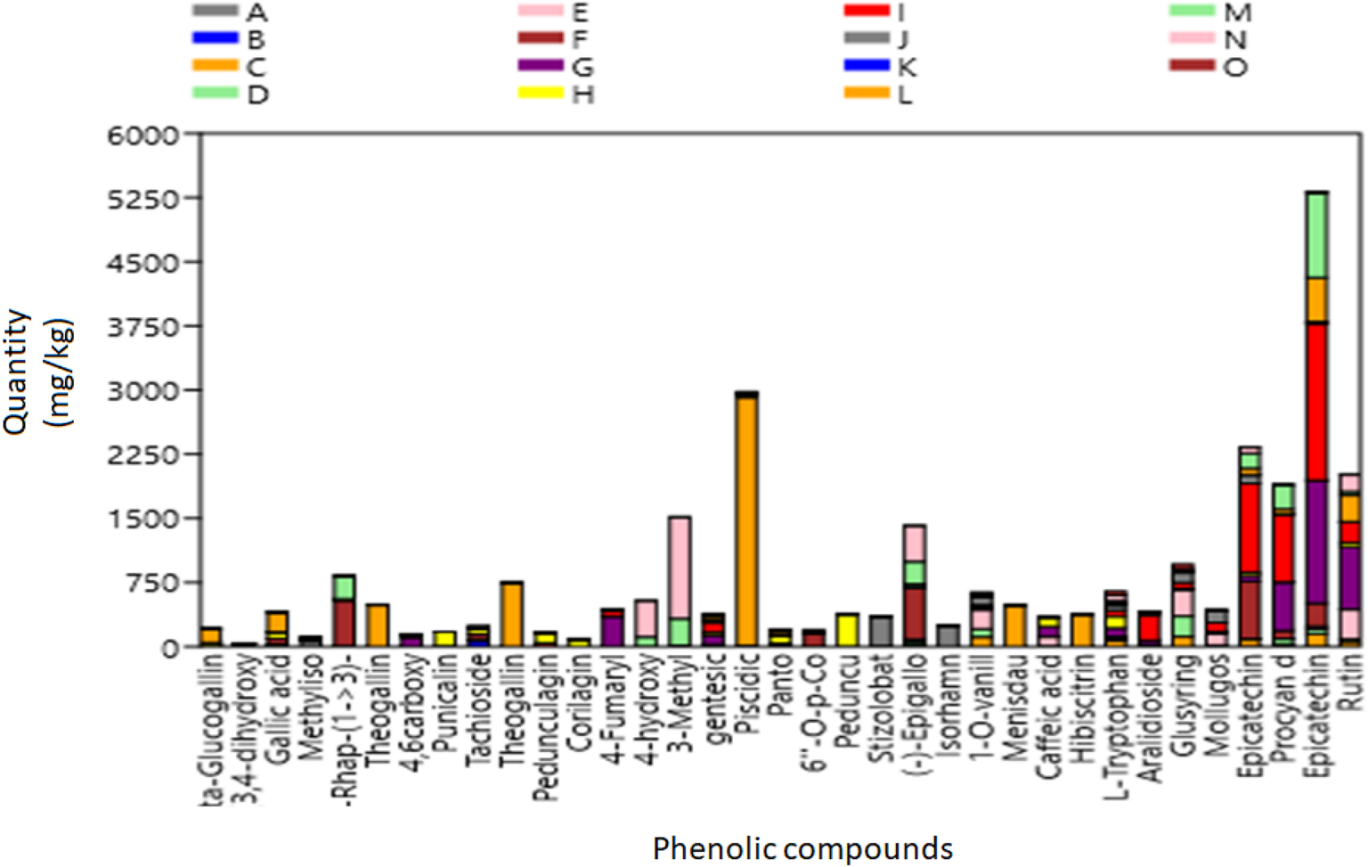
Stacked bar chart of phenolic compounds in different plant species. (***A***) *Aloe esculenta, *(***B***) * Acacia gums, *(***C***) * Acacia nilotica branch, *(***D***)* Boscia albitrunca tuber, *(***E***) * Boscia albitrunca leaves, *(***F***)* Combretum imberbe roots, *(***G***)* Colophospermum mopane leaves, *(***H***) *Combretum imberbe leaves, *(***I***) *Colophospermum mopane bark, *(***J***)* Acacia erioloba bark, *(***K***)* Fockea angustifolia roots, *(***L***)* Ximenia americana leaves, *(***M***)* Ziziphus mucronata roots, *(***N***)* Ziziphus mucronata leaves, *(***O***)* Salvadora persica bark*.

Flavonoids (epicatechin, (-)-Epigallocatechin, and rutin) dominated the distribution of phenolic compounds in the studied plant species, as shown in a stacked bar graph. The phenolic compound 2R, 3S-Piscidic acid was also dominant (Fig. 1).

Beta-Glu; dihydrox; 6- {[2-(3,4-dihydroxyphenyl, Methyliso; Methylisocitric acid, Rhap-(1- > 3); alpha-D-Rhap-(1- > 3)-alpha-D-Rhap, 4,6-O-[(1R)-1-carboxy; 4,6-O-[(1R)-1-carboxyethylidene]-alpha-D-galactose. 4-Fumaryl; 4-Fumarylacetoacetic acid, 4-hydroxy; 4-hydroxy-2-oxoheptanedioate, 3-Methyl; 3-Methylglutaconic acid, Piscidic; (2R,3S)-Piscidic acid, Panto; Pantothenic acid, 6''-O-p-Co; 6''-O-p-Coumaroyltrifolin, Peduncu; Pedunculagin, (-)-Epigallo; (-)-Epigallocatechin, Isorhamn; Isorhamnetin 3-(6''-p-coumarylglucoside), 1-O-vanill; 1-O-vanilloyl-beta-D-glucose, Menisdau; Menisdaurin, Glucosyring; Glucosyringic acid; Syringin 4-O-beta-glucoside.

**Figure 2:**
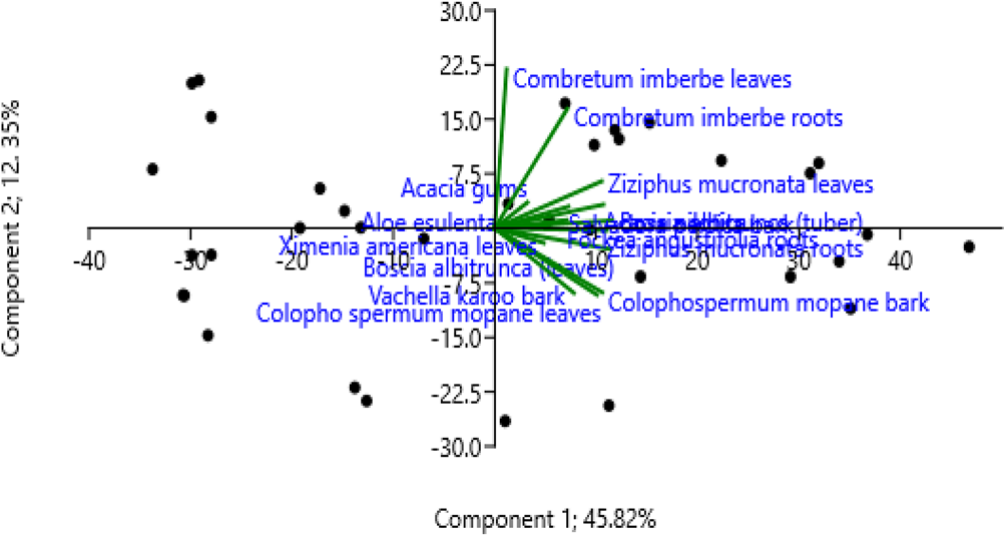
Principal component analysis (PCA) scatter plot of the plant species.

Figure 2 depicts a scatter plot of plant species derived from principal component analysis (PCA). To discover probable variations between plant species, the methods of Hammer et al.^23^ were used. Compared to *A. esculenta*, *A. erioloba*, *F. angustifolia*, *X. americana*, *S. persica*, and *B. albitrunca*, which appear on the left side of the plot, *C. imberbe* leaf and root, *C. mopane* bark, *A. nilotica* branch, and *Z. mucronata* leaf extracts cluster on the right top and bottom side of the plot. In the model shown, the principal components (PCs) explained 58.17% of the variation. The two principal components PC1 and PC2 have 45.85 and 12.35% variability, respectively.

**Figure 3:**
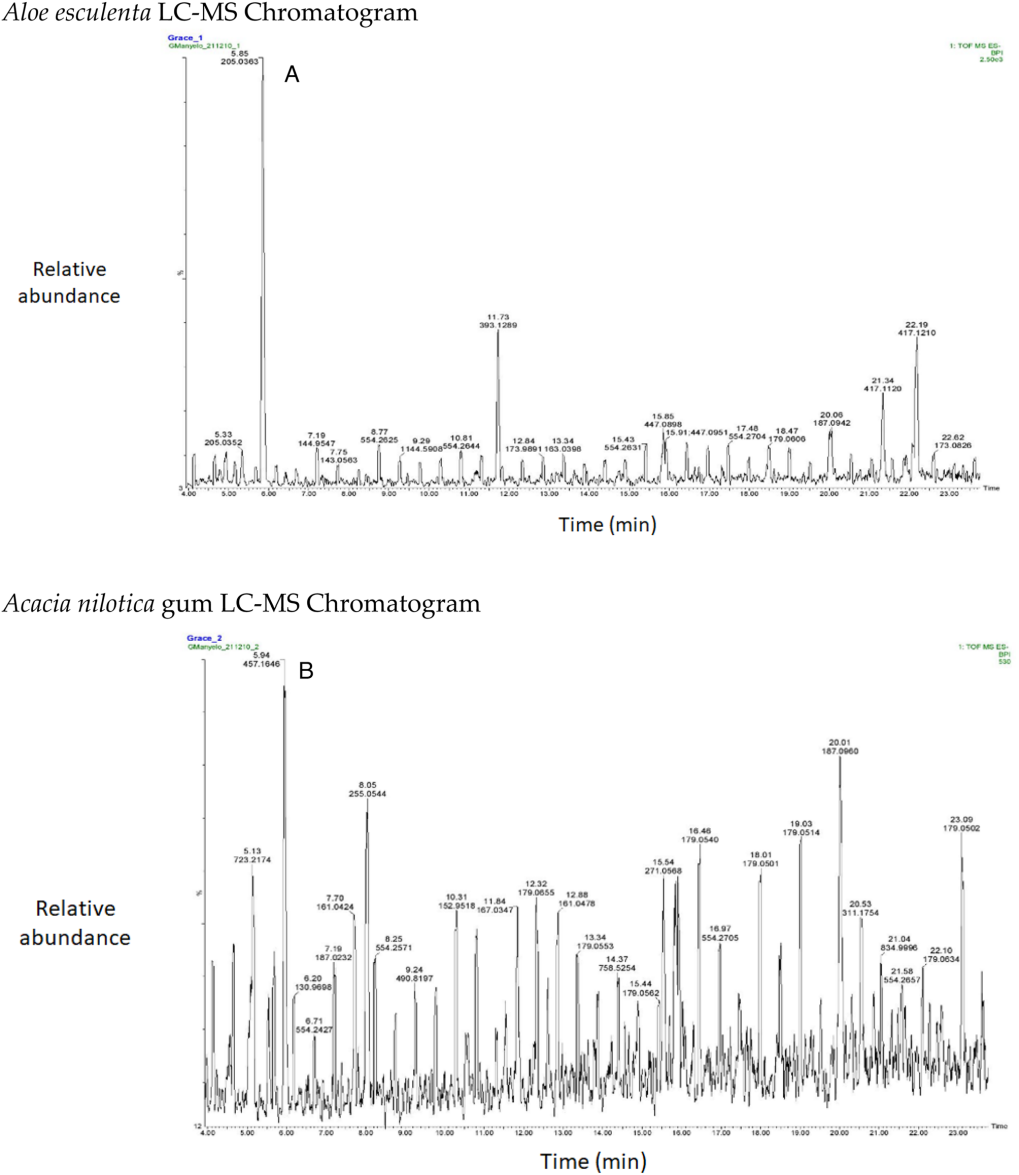

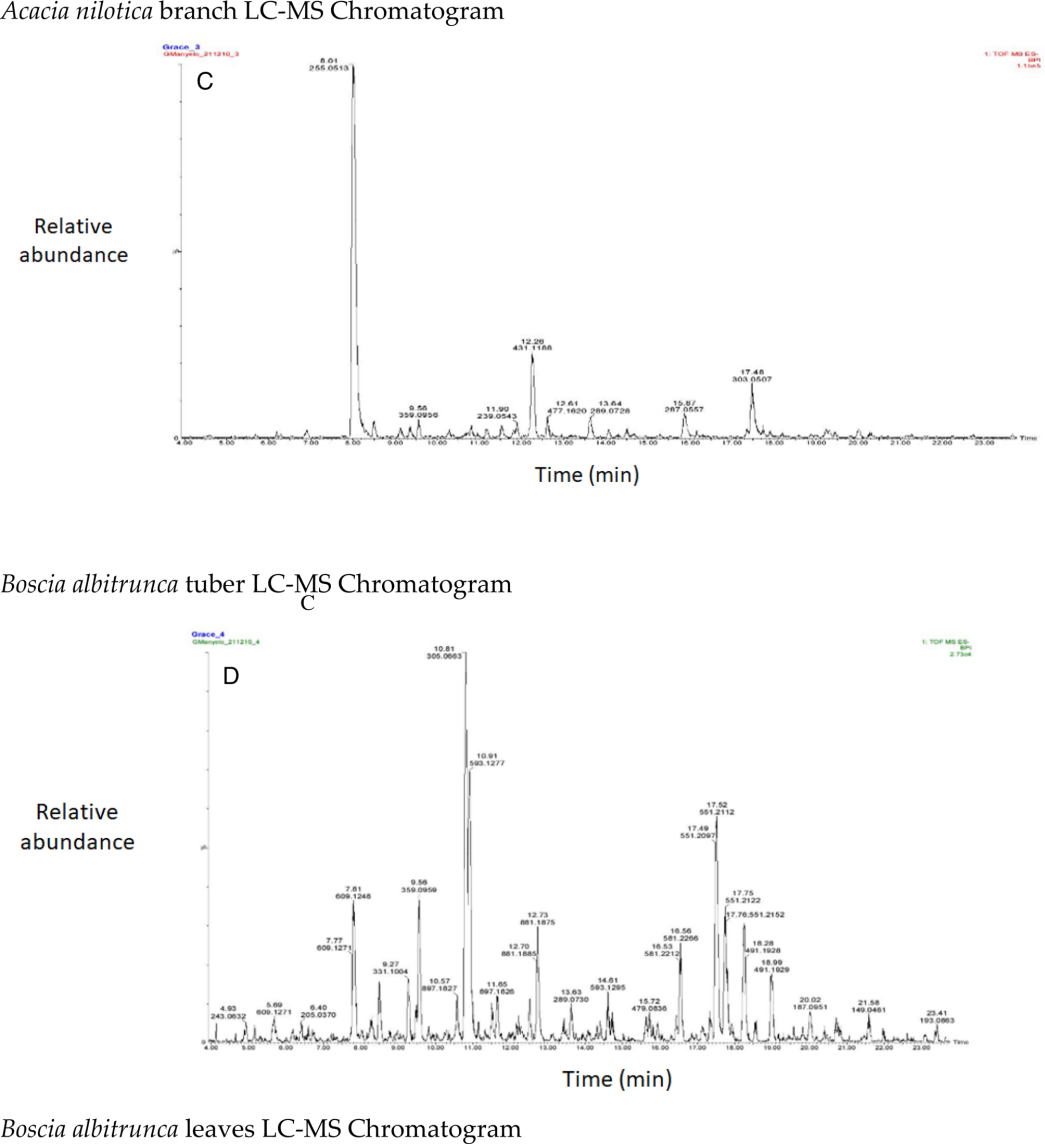

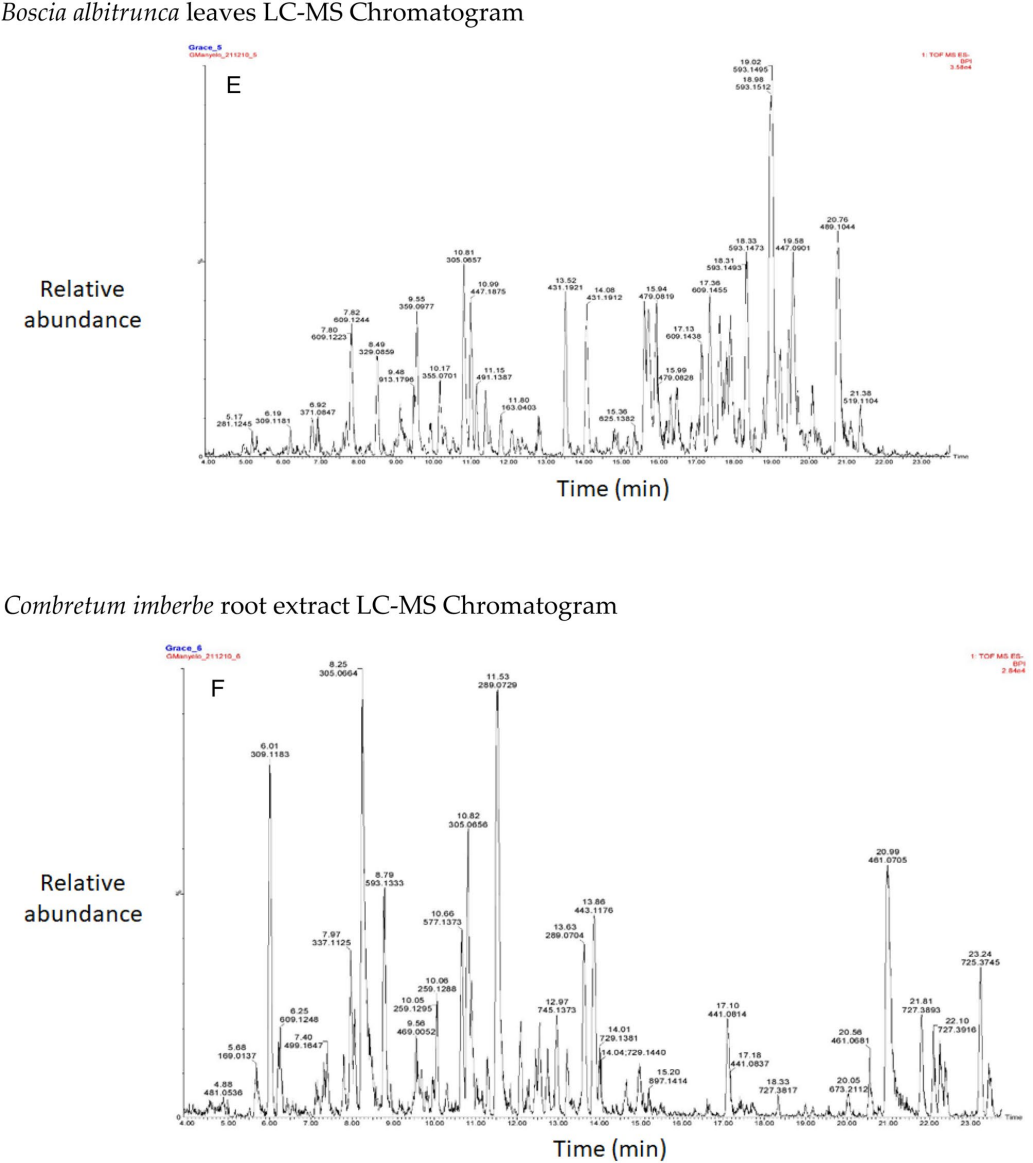

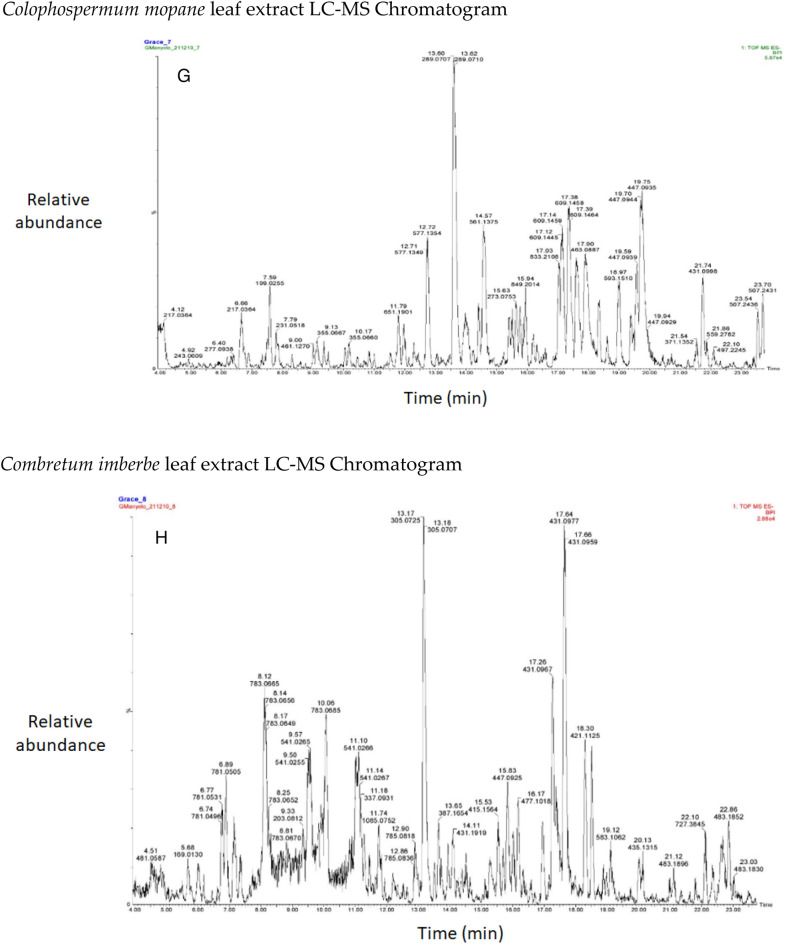

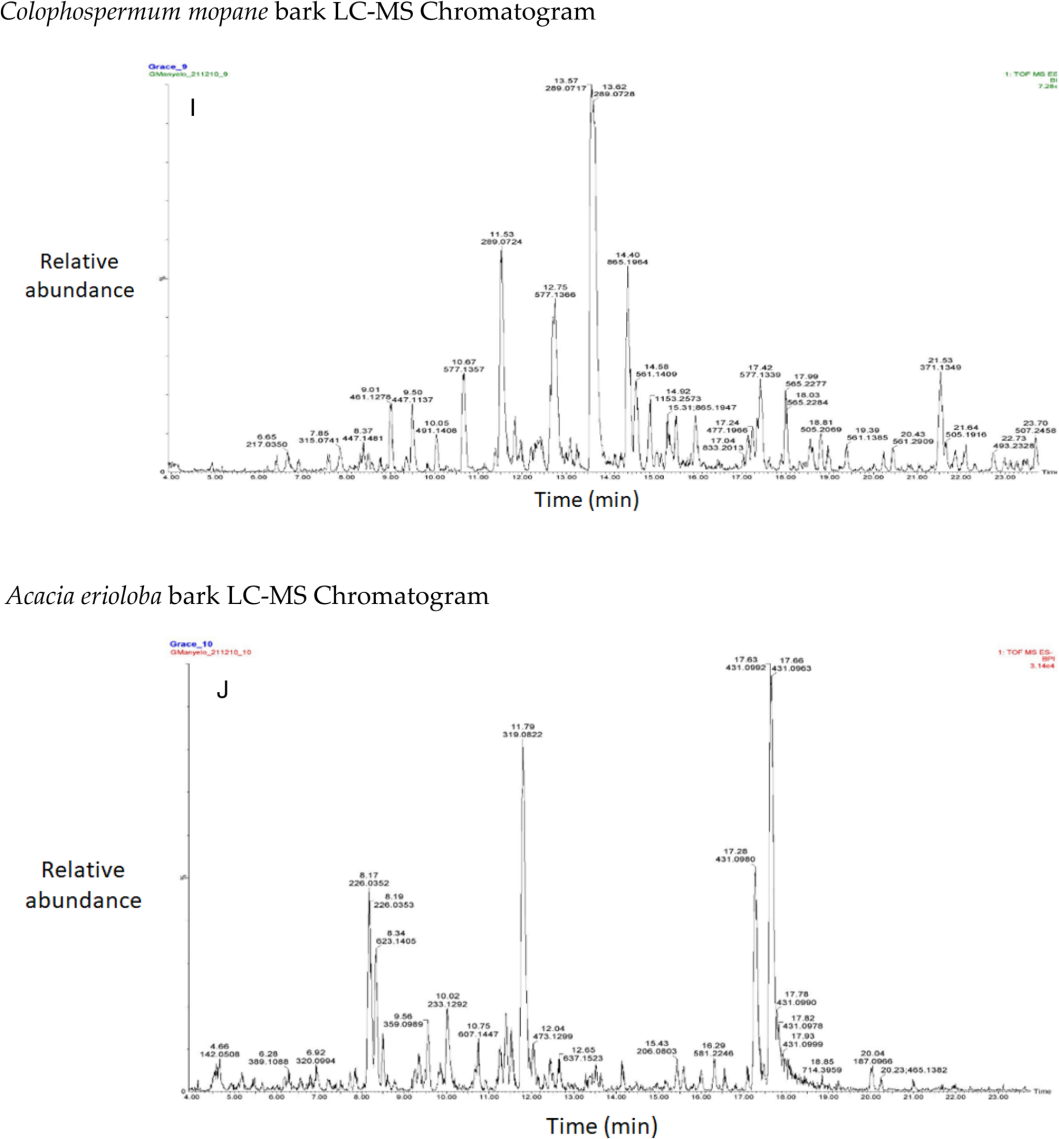

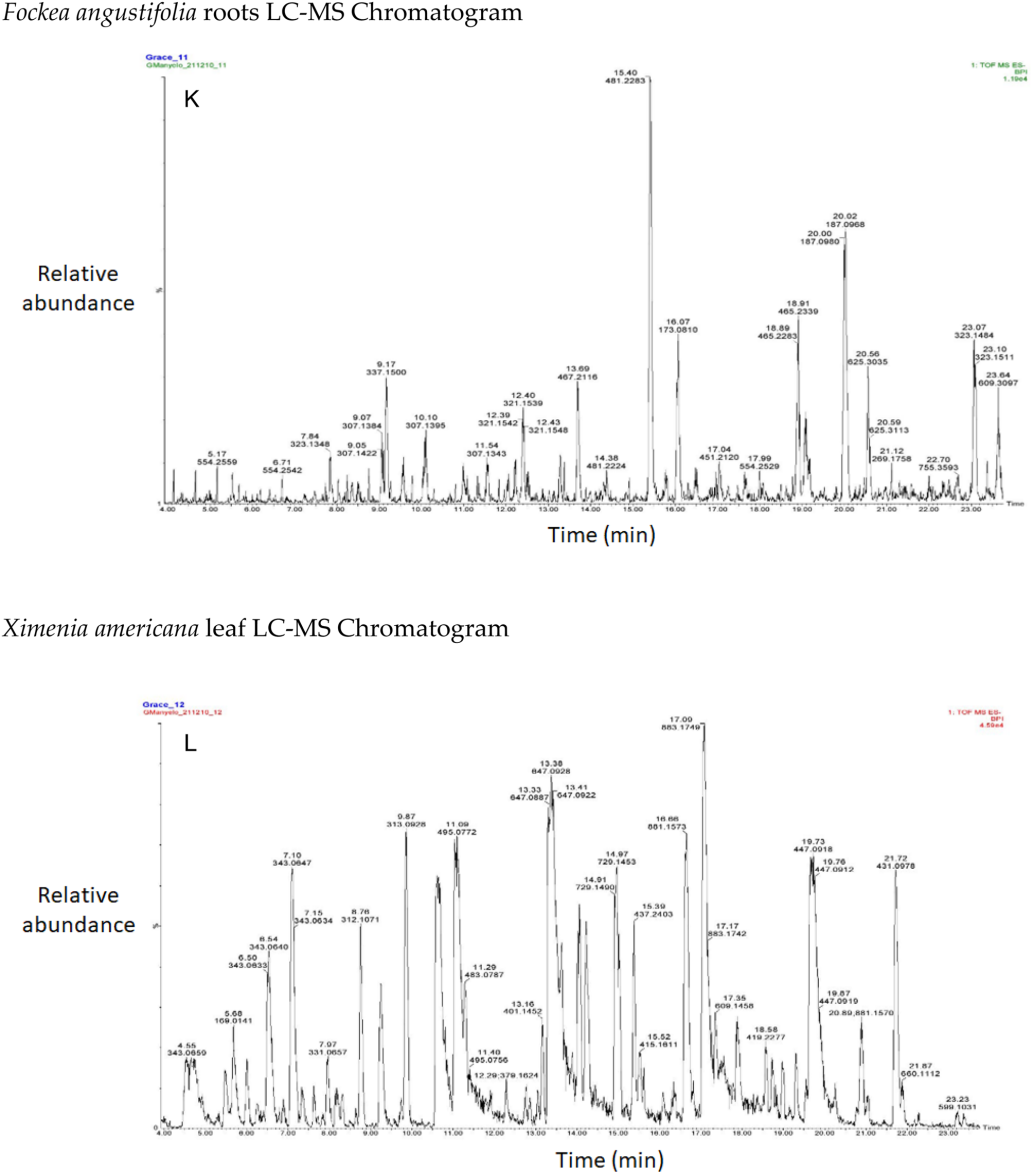

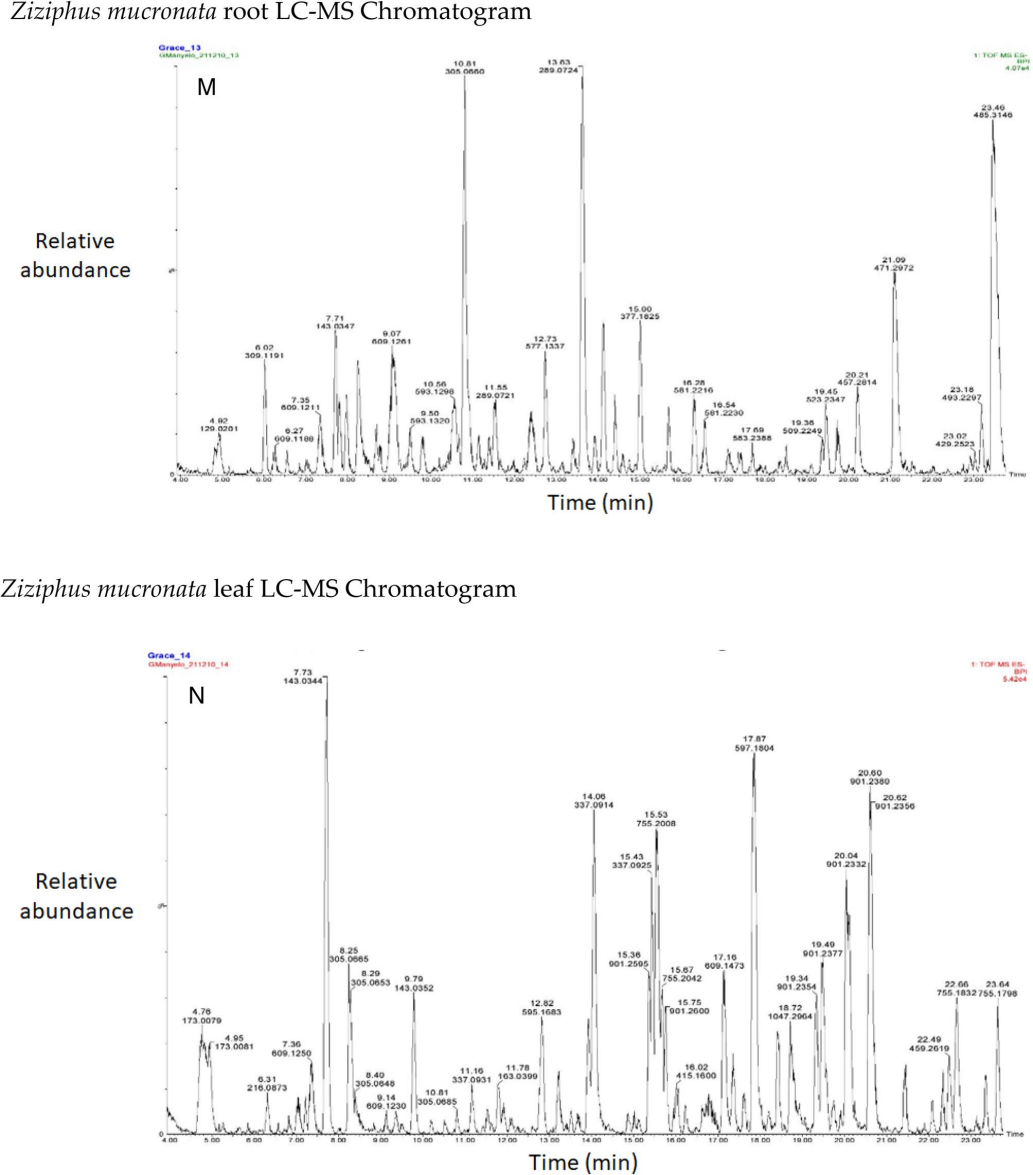

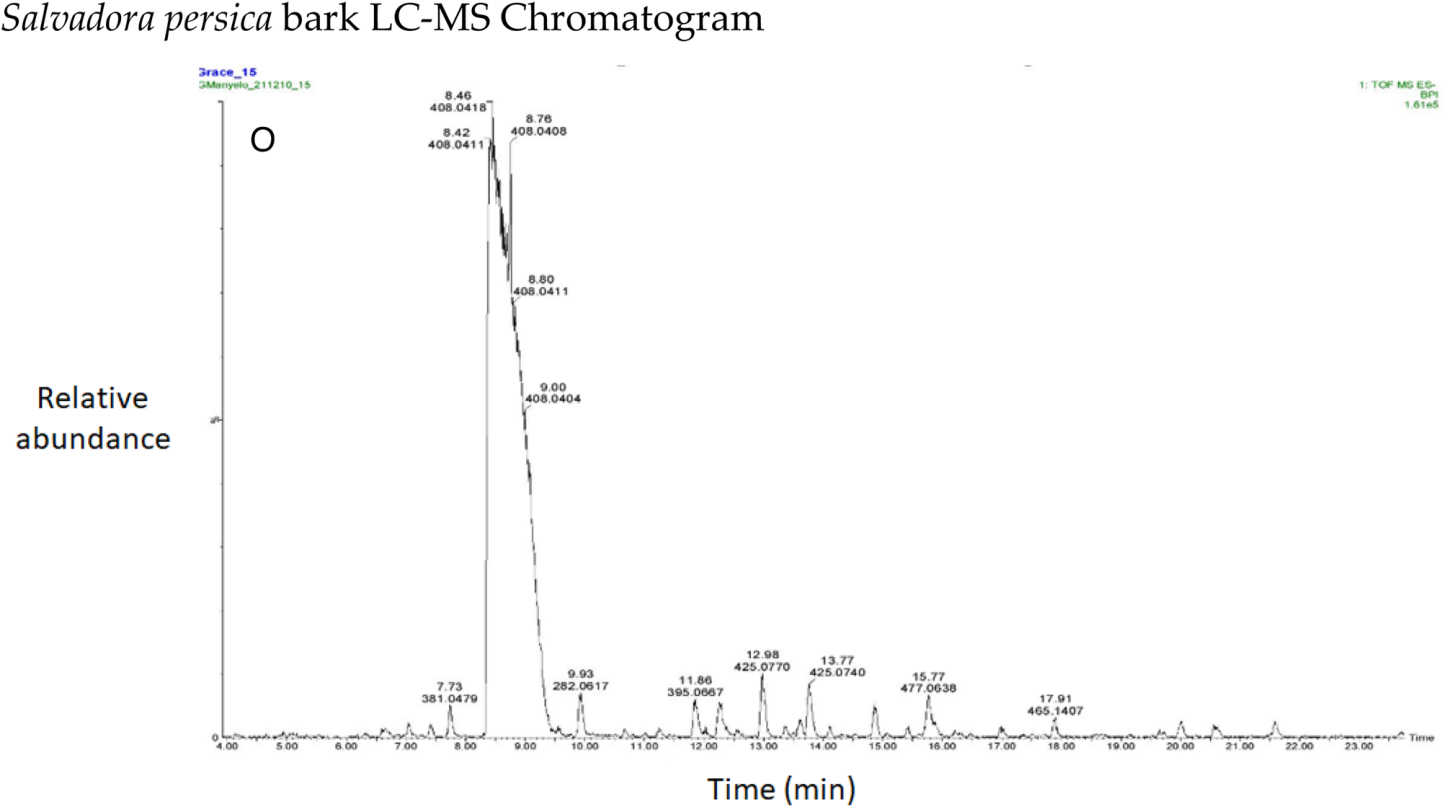
Chromatograms of phenolic compounds in species screened (**A**-**O**). *Aloe esculenta LC–MS Chromatogram*. *Acacia nilotica gum LC–MS Chromatogram*. *Acacia nilotica branch LC–MS Chromatogram*. *Boscia albitrunca tuber LC–MS Chromatogram*. *Boscia albitrunca leaves LC–MS Chromatogram*. *Combretum imberbe root extract LC–MS Chromatogram*. *Colophospermum mopane leaf extract LC–MS Chromatogram*. *Combretum imberbe leaf extract LC–MS Chromatogram*. *Colophospermum mopane bark LC–MS Chromatogram*. *Acacia erioloba bark LC–MS Chromatogram*. *Fockea angustifolia roots LC–MS Chromatogram*. *Ximenia americana leaf LC–MS Chromatogram*. *Ziziphus mucronata root LC–MS Chromatogram*. *Ziziphus mucronata leaf LC–MS Chromatogram*. *Salvadora persica bark LC–MS Chromatogram*.

The chromatograms of the species studied are shown in alphabetical order in Fig. 3A–O. The extracts with multiple peaks included *A. nilotica* gum (Fig. 3A), *A. esculenta* leaf (Fig. 3B), *B. albitrunca* tuber and leaf (Fig. 3D and E), *C. imberbe* root and leaf (Fig. 3F and H); *C. mopane* leaf and bark (Fig. 3G and I); *A. erioloba* bark (Fig. 3J); *F. angustifolia* root (Fig. 3K); *X. americana* leaf (Fig. 3L); and *Z. mucronata* root and leaf (Fig. 3M and N). There were a few peaks in the extracts of A. nilotica branch (Fig. 3C) and S. persica bark (Fig. 3O).

Gallic acid was found in high concentrations in the *X. americana* species, with a retention time of 5.699 min and an Mz of 169,0145 (Table 3). Theogallin was also prominent in *X. americana* species, with a retention time of 6.538 min and an Mz of 343,06,396. Punicalin was mostly found in the leaves of *C. imberbe*, with a retention time of 6.891 min and an Mz of 781,05,054 (Fig. 3H). *Combretum imberbe* leaves were also found to contain (-)-epigallocatechin with a retention time of 13.208 and an Mz of 305,07,166. As illustrated in Fig. 3H, genistin is one of the highest peaks discovered in *C. imberbe* leaves, with a retention time of 17.644 min and an Mz of 431,09,747. Polydine chemical was likewise abundant in *C. imberbe* leaves, with an Mz of 421,11,249 and a detection time of 18.299 min. At a retention time of 15.311 min and an Mz of 865,1947, significant amounts of procyanidin C1 were found in *C. mopane* bark.

*Local name: Oshi, Oshiwambo; Otji, Otjiherero; Kkg, Khoekhoegowab.

## Discussion

The major phytochemical compounds from plants with antioxidant capabilities are known as phenolics and flavonoids^24–27^. For instance, the high concentration of the phenolic compound catechins in green tea has been linked to its antibacterial properties^28,29^. Similarly, epicatechin, a phenolic compound, was said to be responsible for *H. patens*' antibacterial action^30^. The effects of gallic acid on wound healing have also been extensively documented by Pal et al.^31^ and Comino-Sanz et al.^32^. The discovery of a high quantity of gallic acid in *X. americana* may help to explain why this plant is reportedly used in EVM to treat wounds in the current study area. Intriguingly, the current study found evidence of gallic acid, catechin, and epicatechin in the examined species. Both plants are known to be used in EVM to treat a variety of veterinary conditions, including mastitis, wound healing, inflammation, and diarrhoea.

The high levels of 2R,3S-Piscidic acid detected in *A. nilotica* branch extract is compatible with Sulaiman and Balachandran’s^33^ findings, which asserted that *A. nilotica* is the greatest source of phenolics. Furthermore, Ali et al.^34^, reported that *A. nilotica* has a wide variety of biologically active, therapeutically useful compounds that have been used in traditional systems of medicine to treat a variety of ailments. When compared to previous investigations, the current study found *Z. mucronata* leaf extract to contain higher concentration of 3-methyl (3-Methylglutaconic acid) phenolic compound however researchers Gao et al.^35^ and Abdoul-Azize^36^ found smaller amounts of 3-methyl (3-Methylglutaconic acid) in Ziziphus genus samples. *Ziziphus lotus* and *Ziziphus mauritiana* samples, respectively, were found to contain rare phenolic compounds such as chlorogenic acid, 1,3-di-O-caffeoyquinic acid, luteolin-7-o-glucoside, naringin, and kampherol, caffeic, and salviolinic acids^37^.

The differences in phenolic compound presence between aerial and terrestrial organs as detected in species examined are in line with earlier studies by Lattanzio^8^ and Kikowska et al.^38^. Furthermore, Generalić et al.^39^ also revealed seasonal fluctuations in phenolic compound content, while Maina et al.^40^ discovered phenolic compound alterations at different stages of plant development. The pattern of secondary metabolites in each plant is complex^41^; it varies by tissue and organ; regular differences can be seen between developmental stages, for example, organs important for survival and reproduction have the highest and most potent secondary metabolites, and between individuals and populations. Lattanzio^8^ also asserted that there seems to be a correlation between temperature and the concentrations of phenolic compounds in plant tissues, with lower temperatures being linked to higher concentrations of phenolic compounds. Tak and Kumar^42^ claimed that phenolics accumulate in the central vacuoles of guard cells and epidermal cells, as well as subepidermal cells of leaves and shoots, which could explain why *Z. mucronata* and *C. mopane* leaves for example contained more 3-Methyl (3 Methylglutaconic acid), (-)-epigallo (-)-Epigallocatechin, 4,6carboxy, and 4-Fumaryl Fumaryl (4-Fumarylacetoacetic acid) than root and bark extracts. Additionally, since Phenylalanine Ammonia Lyase (PAL) is a key enzyme in the biogenesis of numerous phenolic compounds, its enhanced activity at lower temperatures may help to explain the elevated quantities of phenolic compounds in the leaves than in roots^43^. Similar results were found by Del Valle et al.^44^ who discovered that *S. littorea*'s response to UV stress includes an increase in the concentration of total phenolic compounds. They also discovered that exposure to UV radiation caused a generalized increase in the concentration of anthocyanin and UV-absorbing compounds in *S. littorea*. It is interesting to note that most of the phenolic compounds found in the plant species studied have clustered on principal component 1 (PC1). However, no comparable findings could be found in the literature.

Due to Namibia's frequent droughts, plants have developed several defence mechanisms to deal with or resist this stress^45^. One of these methods is the production of phenolic compounds (polyphenols)^46^. Several native species of severe habitats manufacture phenolic compounds (or polyphenols) as one of their defence mechanisms against the oxidative damage caused by dehydration^47^. The findings of Albergaria et al.^48^ support this, demonstrating that medicinal plants cultivated under severe drought had high amounts of phenolic and flavonoid compounds in the leaves, but those grown under higher irrigation had high levels of phenolic compounds in the seeds. These could explain why the phenolic compounds found in the plant species under investigation varied significantly.

Even though the current study found a remarkable composition of phenolic compounds in the plants under investigation, this does not necessarily mean that the screened plant extracts are effective medications or candidates for drug development. However, it does provide basic information about the plant's phenolic compounds, which are thought to play a significant role in the treatment of veterinary diseases as natural antioxidants, antimicrobials, and antiparasitic agents. More testing of these extracts against pathogenic strains of test organisms will be required to assess the pharmacological efficiency of these plants.

## Conclusions

The overall findings of the present study would act as a standard for subsequent investigations into the pharmacological potentials of plants utilized as ethnoveterinary remedies. Priority should be given to isolating, purifying, and defining the active compounds responsible for these plants’ activity.

## Data Availability

This article contains all data generated or analysed during this study.
